# Characterization of the complete mitochondrial genome of *Pseudocrossocheilus liuchengensis* (Cypriniformes: Cyprinidae)

**DOI:** 10.1080/23802359.2018.1467216

**Published:** 2018-04-28

**Authors:** Bowen Liu, Guangping Cheng, Xiaofeng Shi, Qijia Zhou, Xiuli Chen, Xiaoye Wang, Man Zhang

**Affiliations:** a Guangxi Colleges and Universities Key Laboratory of Aquatic Healthy Breeding and Nutrition Regulation, College of Animal Science and Technology, Guangxi University, Nanning, People’s Republic of China;; b Laboratory of Marine Biology and Ecology, Third Institute of Oceanography, State Oceanic Administration, Xiamen, People’s Republic of China;; c Key Laboratory of Environment Change and Resources Use in Beibu Gulf (Guangxi Teachers Education University), Ministry of Education, Nanning, China;; d Guangxi Academy of Fishery Sciences, Nanning, People’s Republic of China

**Keywords:** *Pseudocrossocheilus liuchengensis*, mitogenome, phylogenetic analysis

## Abstract

*Pseudocrossocheilus liuchengensis* is a Labeoninae fish species endemic to China. In this study, the complete mitochondrial genome of *P. liuchengensis* was firstly determined and described. It was 16,600 bp in length and composed by 13 protein-coding genes, 22 tRNA genes, 2 rRNA genes, and a control region. The overall nucleotide composition was 32.0% of A, 15.5% of G, 25.8% of T, and 26.7% of C, with the AT-rich feature (57.8%). Phylogenetic analysis validated the taxonomic status of *P. liuchengensis*, exhibiting the closest relationship with *P. bamaensis*.

*Pseudocrossocheilus liuchengensis*, belongs to the subfamily Labeoninae, within the family Cyprinidae of the order Cypriniformes (Zhou and Zhang 2005). It is an endemic species to the central and eastern Yunnan-Guizhou Plateau of China, which only distributes in karst underground rivers in Guangxi (Su et al. 2003). Although there are some taxonomic studies about this species (Zhang and Chen 1997; Su et al. 2003), its available mitogenomic information is still limited. In the present study, we provided the first characterization of the complete mitochondrial genome of *P. liuchengensis*, contributing to aid further phylogenetic and genetic studies of this species.

Specimens of *P. liuchengensis* were collected from the underground river located in Du’an autonomous county of Guangxi, China (23°55′6.04″N, 108°01′30.55″E). Muscle samples were fixed in 95% ethanol and preserved in Guangxi Colleges and Universities Key Laboratory of Aquatic Healthy Breeding and Nutrition Regulation, Guangxi University. The DNA extraction was performed using Genomic DNA Isolation Kit (QiaGene, Germany), according to the manufacturer’s instructions. The mitogenome of *P. bamaensis* (GenBank accession no. MF401443) was employed as the reference sequence (Zhang et al. 2017). Twenty-four primers were designed to amplify the PCR products for sequencing. Mitochondrial genome was assembled by DNAman software package (Lynnon Biosoft, Canada), and then annotated using MitoAnnotator (Iwasaki et al. 2013).

The complete mitogenome of *P. liuchengensis* was 16,600 bp in length (GenBank accession no. MF817726) and was AT-biased (57.8%), with a base composition of 32.0% A, 15.5% G, 25.8% T, and 26.7% C. Genomic organization showed high degree of conservation among *P. liuchengensis* and other Labeoninae fishes, including 13 protein-coding genes (PCGs), 22 *tRNA* genes, 2 *rRNA* genes, and a control region (D-loop). The typical start codon ATG appeared in all the coding genes except in *COI* gene, having GTG as the initiation codon. Seven PCGs used a common stop codon of TAA, whereas the remaining six were terminated by incomplete stop codon T or TA. The length of 22 *tRNAs* ranged from 66 bp (*tRNA^Cys^*) to 76 bp (*tRNA^Leu^* and *tRNA^Lys^*). The *12S* and *16S rRNA* genes were 954 and 1688 bp in length, respectively. These two *rRNAs* were located between *tRNA^Phe^* and *tRNA^Leu^*, and spaced by *tRNA^Val^*. The D-loop was located between *tRNA^Pro^* and *tRNA^Phe^*, with a size of 937 bp and a high AT content (66.1%).

To validate the phylogenetic position of *P. liuchengensis*, the Maximum Likelihood tree was constructed by MEGA 7.0 software using 13 mitochondrial PCGs from mitogenomes of *P. liuchengensis*, 19 other Labeoninae fishes, and one outgroup from Barbinae (Kumar et al. 2016). Evolutionary model selection was inferred using PhyML 3.0, where the substitution model GTR + G + I was resulted as the best-fit model (Guindon et al. 2010). As shown in Figure 1, *P. liuchengensis* was positioned as the sister species of *P. bamaensis* within the genus *Pseudocrossocheilus*, which was in accordance with the traditional morphological classification and recent molecular works (Su et al. 2003; Yang et al. 2012). The mitogenomic resource obtained in the present study will provide valuable information for further genetic investigations of *P. liuchengensis*.

**Figure 1.**
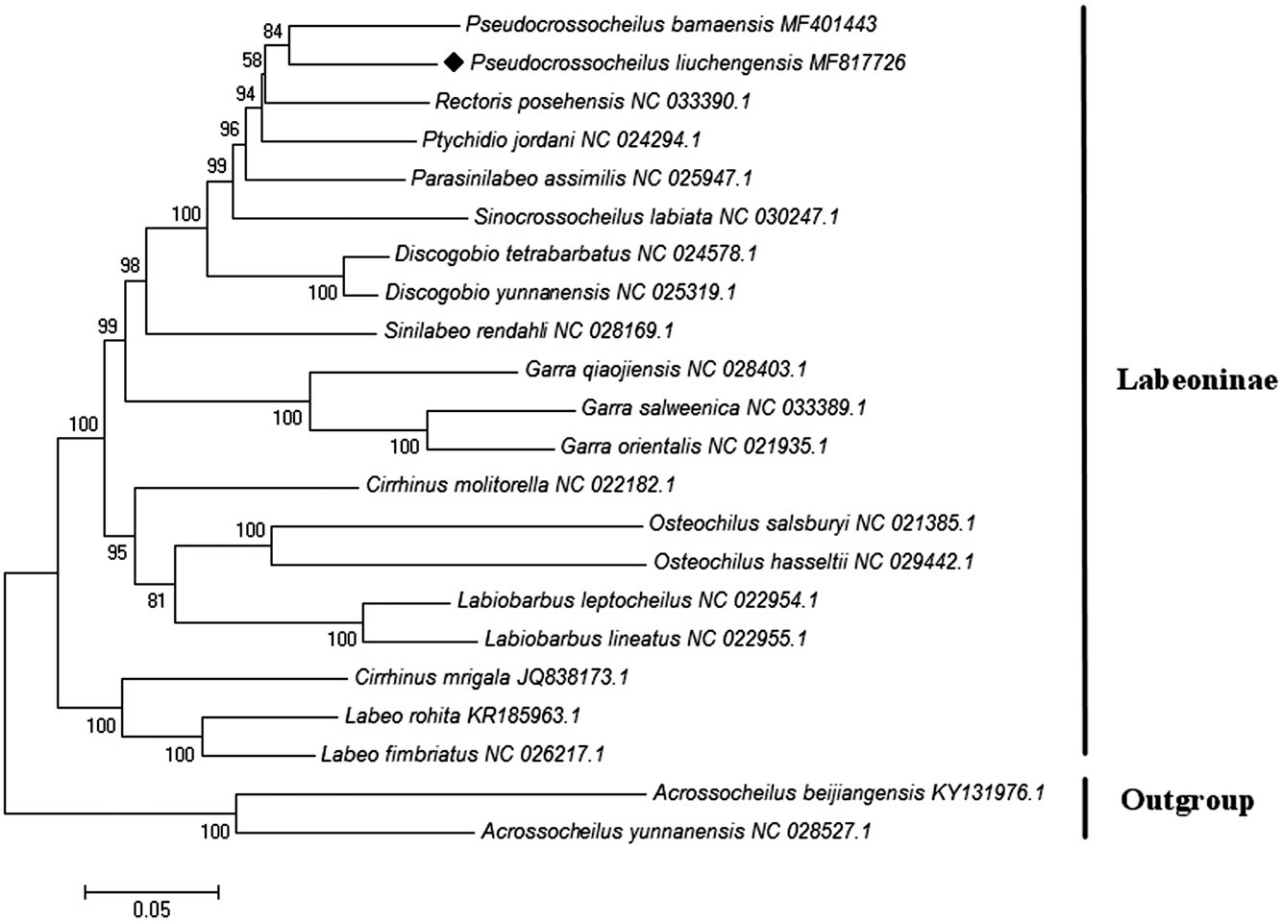
Phylogenetic relationships of *Pseudocrossocheilus liuchengensis*, 19 other Labeoninae fishes and one outgroup from Barbinae based on 13 concatenated mitochondrial PCGs by Maximum Likelihood analysis with GTR + G + I model. The number on branches indicates posterior probabilities in percentage. The number after the species name is the GenBank accession number. The mitogenomic information of *P. liuchengensis* is marked with rhombus.
